# Effects of Reduced Weight Maintenance and Leptin Repletion on Functional Connectivity of the Hypothalamus in Obese Humans

**DOI:** 10.1371/journal.pone.0059114

**Published:** 2013-03-21

**Authors:** William Hinkle, Michael Cordell, Rudy Leibel, Michael Rosenbaum, Joy Hirsch

**Affiliations:** 1 Department of Neuroscience, Columbia University Medical Center/New York Presbyterian Medical Center, New York, New York, United States of America; 2 Institute of Human Nutrition, Columbia University Medical Center/New York Presbyterian Medical Center, New York, New York, United States of America; 3 Department of Pediatrics, Division of Molecular Genetics, Naomi Berrie Diabetes Center, Columbia University Medical Center/New York Presbyterian Medical Center, New York, New York, United States of America; 4 Departments of Radiology and Psychology, Columbia University Medical Center/New York Presbyterian Medical Center, New York, New York, United States of America; 5 fMRI Research Center, Columbia University Medical Center/New York Presbyterian Medical Center, New York, New York, United States of America; University of Missouri-Kansas City, United States of America

## Abstract

Treating obesity has proven to be an intractable challenge, in part, due to the difficulty of maintaining reduced weight. In our previous studies of in-patient obese subjects, we have shown that leptin repletion following a 10% or greater weight loss reduces many of the metabolic (decreased energy expenditure, sympathetic nervous system tone, and bioactive thyroid hormones) and behavioral (delayed satiation) changes that favor regain of lost weight. FMRI studies of these same subjects have shown leptin-sensitive increases in activation of the right hypothalamus and reduced activation of the cingulate, medial frontal and parahippocampal gryi, following weight loss, in response to food stimuli. In the present study, we expanded our cohort of in-patient subjects and employed psychophysiological interaction (PPI) analysis to examine changes in the functional connectivity of the right hypothalamus. During reduced-weight maintenance with placebo injections, the functional connectivity of the hypothalamus increased with visual areas and the dorsal anterior cingulate (dorsal ACC) in response to food cues, consistent with higher sensitivity to food. During reduced-weight maintenance with leptin injections, however, the functional connectivity of the right hypothalamus increased with the mid-insula and the central and parietal operculae, suggesting increased coupling with the interoceptive system, and decreased with the orbital frontal cortex, frontal pole and the dorsal ACC, suggesting a down-regulated sensitivity to food. These findings reveal neural mechanisms that may underlie observed changes in sensitivity to food cues in the obese population during reduced-weight maintenance and leptin repletion.

## Introduction

Obesity has become the most prevalent and costly nutritional problem in the United States and currently accounts for more than 10% of direct U.S. health care spending [1]. Modest (10%) weight loss is sufficient to prevent or ameliorate many of the major medical and metabolic consequences of obesity [2]. While most patients can achieve such weight loss by conventional means, the majority cannot maintain the reduced weight for extended periods of time [3].

Our previous work has shown that maintenance of a reduced body weight is accompanied by disproportionately decreased rates of energy expenditure, largely attributable to increased skeletal muscle work efficiency as well as decreased sympathetic nervous system tone and circulating concentrations of bioactive thyroid hormones and increased parasympathetic nervous system tone [4]–[6]. This disproportionate decline in energy expenditure (∼300–400 kcal/day below the total predicted solely on the basis of weight and body composition changes) would have little consequence if it were easy to sustain a corresponding reduction in energy intake in order to maintain a reduced body weight. However, we [7], [8] and others [9]–[11] have shown, this is not the case.

**Figure 1:**
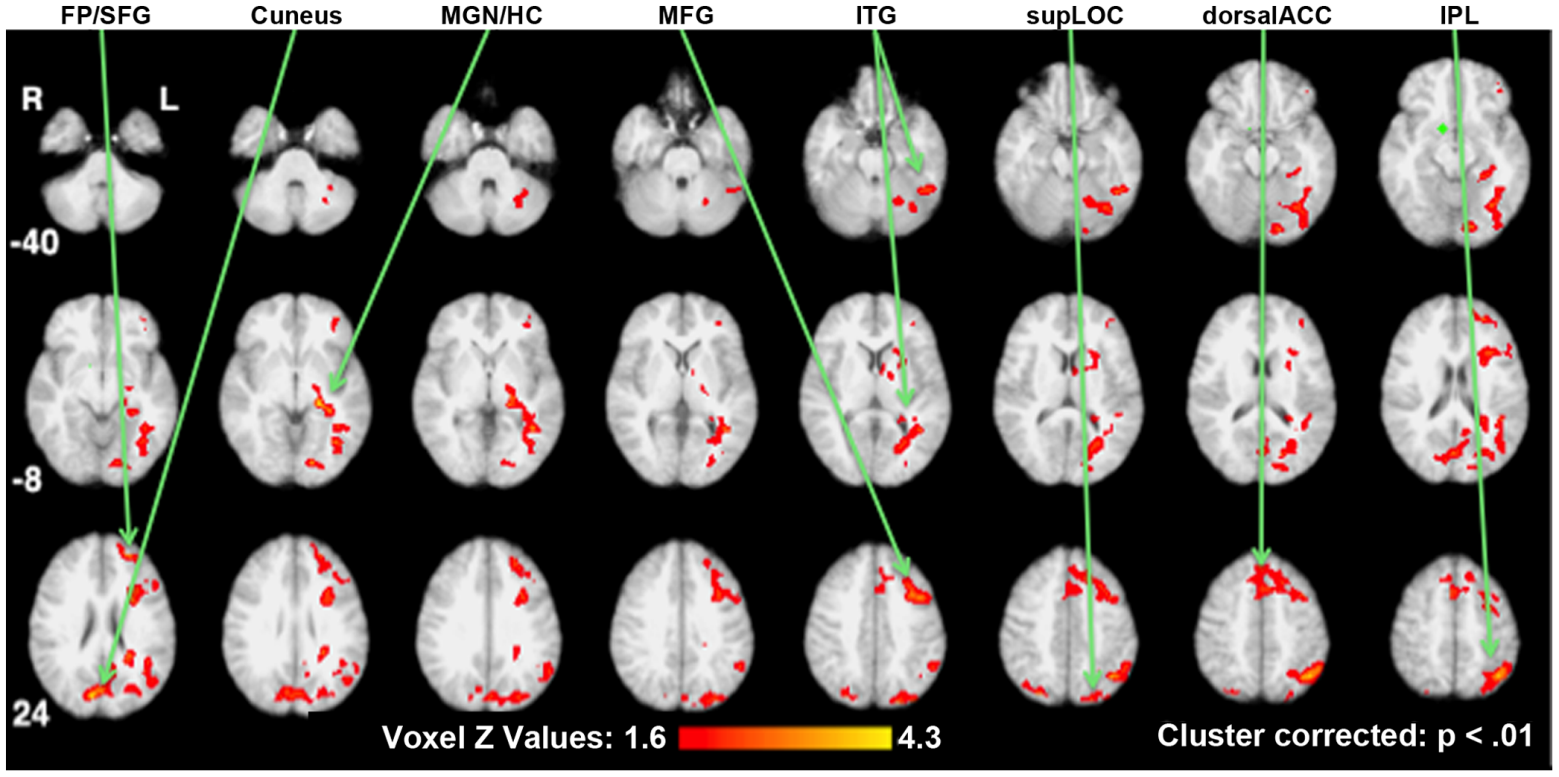
Increased functional connectivity in the reduced-weight maintenance with placebo injections comparison. Brain areas showing significant increases in functional connectivity with the hypothalamic seed (indicated in green, upper right) are shown on standard space axial brain slices with the color indicating the Z score per the color gradient on the bottom. FP (Frontal Pole), SFG (Superior Frontal Gyrus), MGN (Medial Geniculate Nucleus), HC (Hippocampus),MFG (Middle Frontal Gyrus), ITG (Inferior Temporal Gyrus), supLOC (superior division of the Lateral Occipital Cortex), dorsal ACC (dorsal Anterior Cinqulate Cortex), IPL (Inferior Parietal Lobule).

Our previous fMRI investigation in a population of in-patient obese subjects [8] compared responses to visual food versus non-food cues in a population of in-patient obese subjects in three treatment conditions: stabilized at maximal weight (Wt_initial_), following stabilization at 10%–12% below usual weight while receiving twice daily injections of either a placebo (Wt_−10%placebo_) or while receiving twice daily leptin injection in doses titrated to restore 8 a.m. leptin concentrations to those present prior to weight loss (Wt_−10%leptin_) (See Figure 1). We found that during maintenance of a 10% or greater reduced body weight, there are increases in neural activity of brain areas that are associated with reward valuation and processing of food-related stimuli and decreases in neural activity of brain areas related to restraint in response to food [8]. These previous fMRI findings accompanied behavioral studies in the same population demonstrating that individuals maintaining a reduced weight experience delayed satiation, decreased perception of how much food they have eaten and increased hunger [7]. We also found that in the leptin repletion condition, the hypothalamus was more active and the cingulate, medial frontal and parahippocampal gryi were less active in response to food cues [8]. These variations in activity patterns suggest variations in functional connectivity that were tested in the present study to advance understanding of the neural dynamics associated with the effects of leptin during body weight fluctuations.

The combination of decreased energy expenditure and dysregulation of systems controlling energy intake during reduced weight maintenance tend to bias physiological responses toward weight regain. Many of these changes in energy homeostatic systems following weight loss are at least partially reversed by exogenous administration of the adipocyte-derived hormone leptin [5], [7], [8]. The previous findings contributed insight into the brain areas that respond differentially to food cues as a result of weight loss and leptin repletion, including the demonstration that leptin repletion reverses the decline in hypothalamic activation following weight loss. Beyond these findings, little is known about how functioning circuits, rather than individual loci, in the brain adapt their responses to food cues in weight loss or leptin treatment.

In the present study, we expanded our in-patient cohort and used psychophysiological interaction (PPI) analysis to examine the functional connectivity between brain areas as subjects were exposed to food cues in the same three treatment conditions. This approach is based upon the understanding that the physiological connections between two or more brain regions vary with the function or psychological context [12]. The benefit of PPI analysis is that it allows the identification of separate brain areas that, under certain psychological conditions (i.e. viewing food compared to non-food cues), form significantly coordinated functional networks. In PPI analysis a “seed” region of interest in the brain is specified and correlations with activations of other brain areas are determined based on the interaction of a psychological regressor (the time course of the external stimuli convolved with a hemodynamic response function) and a physiological regressor (the activation time course of the seed). Areas that show statistically significant correlations are said to be functionally connected (or coupled) to the seed when responding to the external stimulus, e.g. food cues. Based on our previous analyses of reduced weight maintenance and leptin effects on activation of individual brain loci in response to food, we chose the right hypothalamus as the primary seed of interest and tested two hypotheses: First, that the 10% weight reduced condition, compared to the initial weight condition (Wt_−10%placebo_ contrasted with Wt_initial_), would increase functional connectivity of the hypothalamus with the attention and visual systems when viewing food cues compared to non-food cues, as evidence of up-regulated sensitivity to food cues (e.g., increased reward value) during maintenance of reduced weight [13], [14]. Second, that the leptin repletion condition, compared to the reduced weight condition with placebo injections (Wt_−10%leptin_ contrasted with Wt_−10%placebo_), would (a) increase functional connectivity of the hypothalamus with the insula, as evidence of achieving some aspects of the homeostatic coordination within the intrinsic food regulation system [15], [16]; and (b) reduce functional connectivity of the hypothalamus with reward valuation areas when responding to food cues, as evidence of down regulation of extrinsic food cue salience [15]. In addition to our primary hypothalamic seed, we examined the bilateral nucleus accumbens as a secondary or confirmatory seed. Given the pivotal role of the nucleus accumbens in actual energy intake behavior, one would predict similar effects on the changes in functional connectivity observed for both the hypothalamus and nucleus accumbens relative to the neural systems regulating energy intake.

## Results

**Figure 2:**
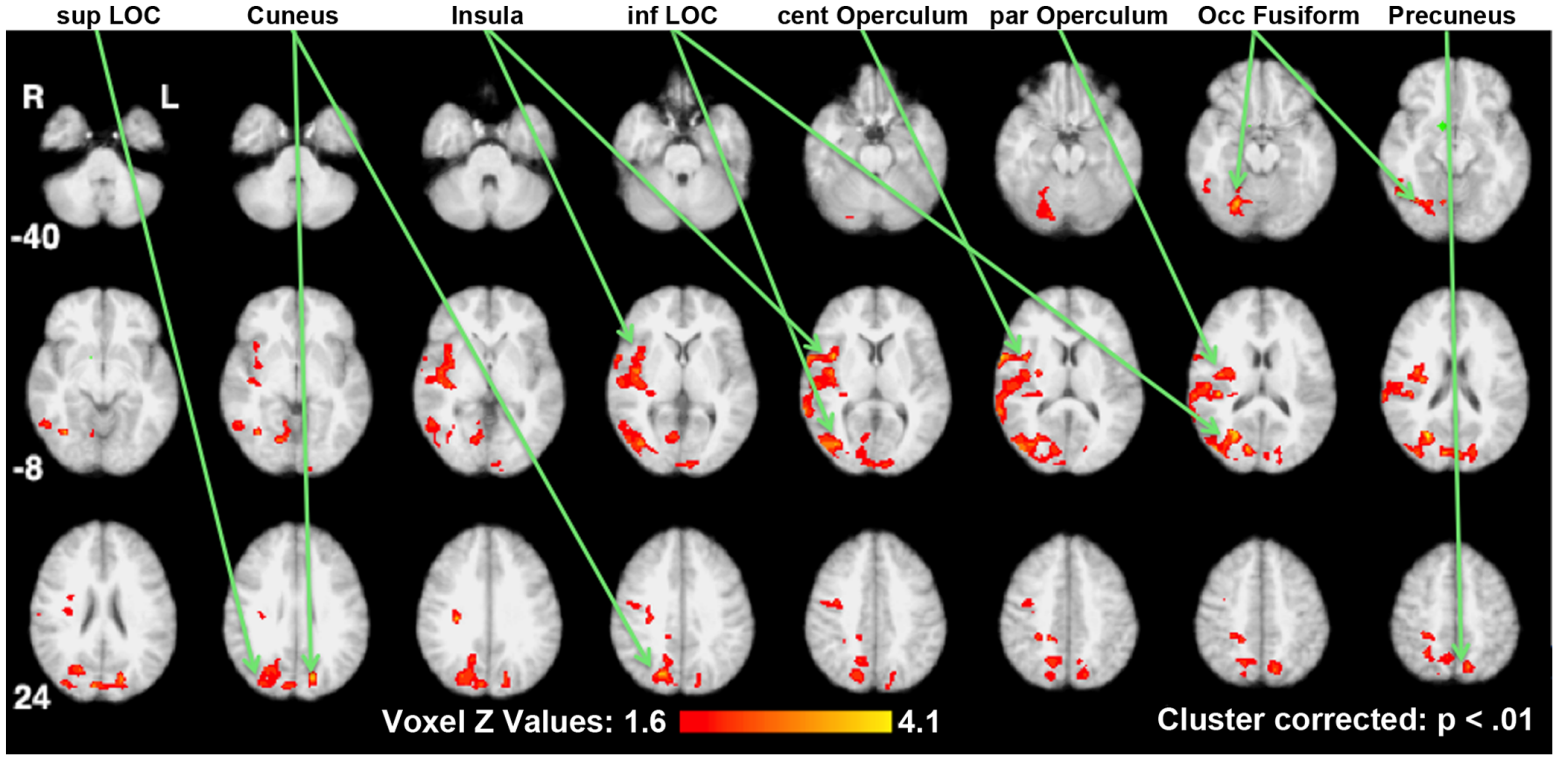
Increased functional connectivity in the reduced-weight maintenance with leptin repletion comparison. Brain areas showing significant increases in functional connectivity with the hypothalamic seed (indicated in green) are shown on standard space axial brain slices with the color indicating the Z score per the color gradient on the bottom. SupLOC (superior division of Lateral Occipital Cortex), inf LOC (inferior division of the Lateral Occipital Cortex).

**Figure 3:**
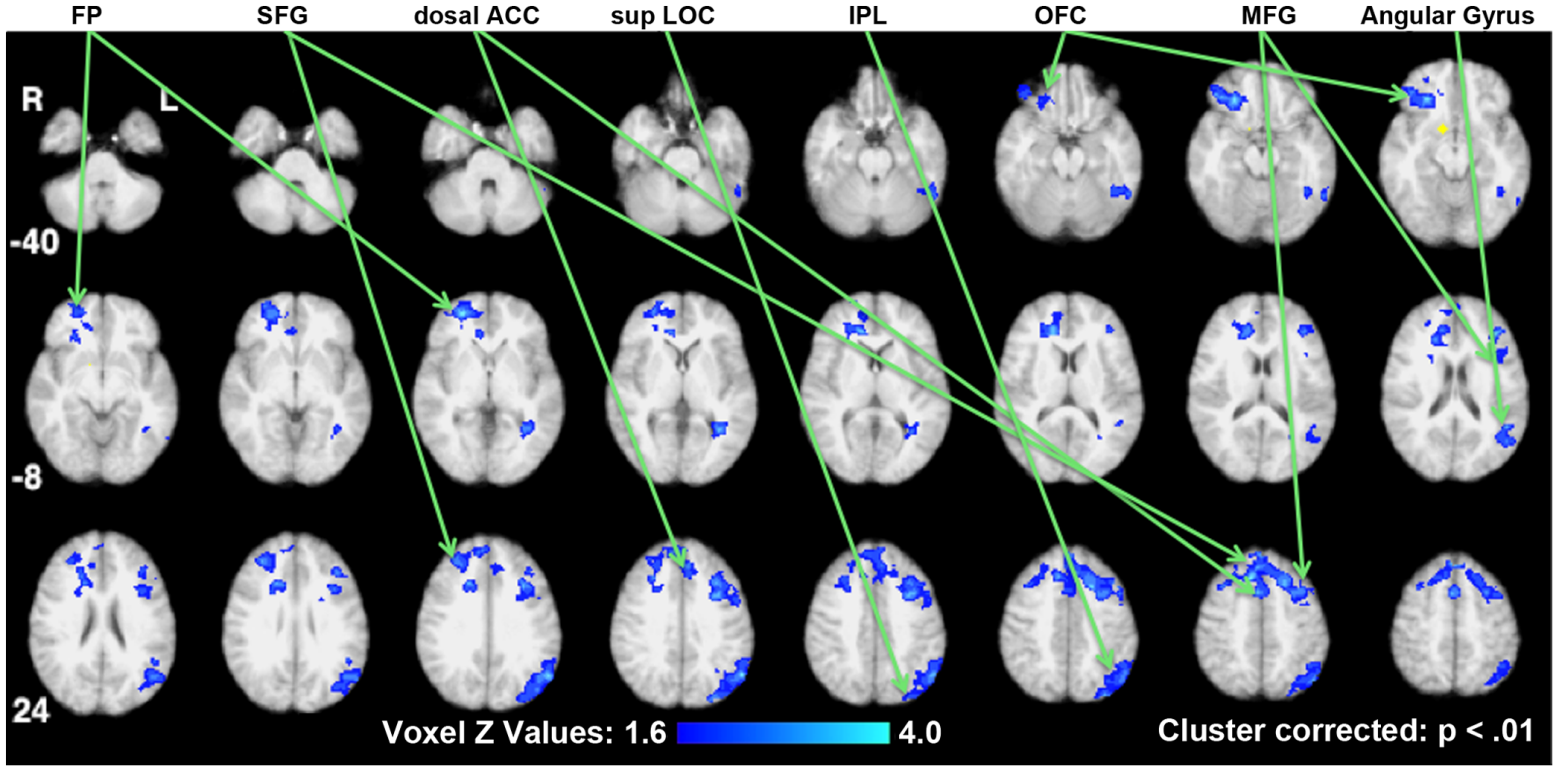
Decreased functional connectivity in the reduced-weight maintenance with leptin repletion comparison. Brain areas showing significant decreases in functional connectivity with the hypothalamic seed (indicated in yellow) are shown on standard space axial brain slices with the color indicating the Z score per the color gradient on the bottom. FP (Frontal Pole), SFG (Superior Frontal Gyrus), dorsal ACC (dorsal Anterior Cingulate Cortex), sup LOC (superior division of the Lateral Occipital Cortex), IPL (Inferior Parietal Lobule), OFC (Orbital Frontal Cortex), MFG (Medial Frontal Gyrus).

PPI analyses were constructed to test the effects of reduced-weight maintenance (i.e. Wt_−10%placebo_ contrasted with Wt_initial_) and leptin repletion (i.e. Wt_−10%leptin_ contrasted with Wt_−10%placebo_) (See Figure S1). Table 1 summarizes changes in functional connectivity for the hypothalamic seed in these two comparisons. For these comparisons, Figures 1, 2, 3 present statistical parametric maps of increases in functional connectivity shown in warm colors and decreases in functional connectivity shown in cool colors, overlaid on normalized axial brain slices ranging from z = −40 mm to z = 52 mm relative to the anterior commissure-posterior commissure line.

**Table 1:** Changes in Functional Connectivity^1^.

Comparison	Structure	Z score	x	y	z
**Weight Loss (Wt_−10placebo_>Wt_initial_)**					
** (See ** **Figure 1** **)**	Significant **increased** functional connectivity relative to food cues:				
	Cuneus	4.35	10	−80	24
	Inf Parietal Lobe	3.75	−38	−62	46
	Inf Parietal Lobe/SPL	3.60	−42	−60	54
	MGN/Hippocampus	3.55	−20	−28	−6
	Inf Parietal Lobe	3.48	−44	−62	48
	MFG	3.17	−30	2	54
	MFG	3.15	−44	10	40
	ITG	3.06	−46	−52	−22
	Frontal Pole/SFG	2.84	−22	50	26
	MFG	2.81	−34	14	40
	sup LOC	2.47	−22	−84	40
	dorsal ACC	2.45	−6	16	46
	No significant decreases in functional connectivity were observed				
**Leptin Repletion (Wt_−10%leptin_>Wt_−10%placebo_)**					
** (See ** **Figure 2** **)**	Significant **increased** functional connectivity relative to food cues:				
	Cuneus	3.41	−14	−76	28
	sup LOC	3.28	44	−80	16
	inf LOC	3.27	38	−76	6
	Precuneous	3.26	20	−74	36
	parietal Operculum	3.64	42	−28	18
	Broca/precentral Gyrus	3.57	62	8	14
	Fusicform	3.48	24	−68	−16
	central Operculum	3.46	40	6	10
	Insula	3.03	42	−8	6
** (See ** **Figure 3** **)**	Significant **decreased** functional connectivity relative to food cues:				
	OFC	4.05	26	32	−16
	SFG/MFG	3.94	−20	20	46
	Frontal Pole	3.92	26	52	0
	SFG	3.80	10	26	48
	MFG	3.60	−38	34	20
	SFG	3.53	0	48	46
	IPL	3.69	−56	−64	34
	sup LOC	3.36	−34	−82	46
	sup LOC	3.32	−48	−68	48
	sup LOC	3.14	−32	−84	34
	Angular Gyrus	3.07	−48	−52	20
	dorsal ACC	2.91	−4	38	36

**Seed: Right Hypothalamus:** Peak voxel location of changes in functional connectivity as a result of condition comparisons as represented by local maxima within clusters. Standard coordinates localized using the Montreal Neurological Institute MNI-152 brain template.

1 Located as local maxima in clusters expressed in the Montreal Neurological Institute 152 brain template.

### Reduced-weight Maintenance Comparison

The changes in the functional connectivity of the hypothalamus as a result of reduced-weight maintenance were determined by contrasting the results of Wt_−10%placebo_ with those of Wt_initial_ and are shown in Figure 1. The right hypothalamus seed area is shown in green (z = −12 mm, upper right). These results reflect an increased sensitivity to visual and attention processing areas relative to food cues.

Increases in functional connectivity (coupling) of the hypothalamus when viewing food stimuli extended to multiple areas that can be broadly grouped as visual, memory and attention areas, as shown in Table 1 and Figure 1. These areas included ventral visual (occipital fusiform and temporal fusiform areas) and dorsal visual (superior lateral occipital cortex (LOC) and cuneus) areas, the hippocampus, and areas associated with attention and executive function systems (dorsal anterior cingulate cortex (ACC) and left middle and inferior frontal gyri). There were no significant decreases in functional connectivity with the hypothalamus, when viewing food cues as a result of reduced-weight maintenance.

### Leptin Repletion Comparison

Changes in functional connectivity of the hypothalamus as a result of the leptin repletion were determined by contrasting the results of Wt_−10%leptin_ with those of Wt_−10%placebo_ and are shown in Table 1 and Figures 2 and 3.

As a result of leptin repletion, there were increases in functional connectivity in response to visual food cues of the right hypothalamus with the right insula cortex, the adjoining central and parietal operculae, and with right-dominant visual areas in the ventral visual stream (occipital fusiform cortex and inferior division of the LOC), and the dorsal stream (the cuneus and the superior division of the LOC) (Figure 2).

There were also decreases in the functional connectivity (decoupling) of the hypothalamus in response to visual food cues during leptin administration compared to placebo, with ventral frontal areas (the right frontal pole and the right orbital frontal cortex) and dorsal frontal areas (superior frontal gyrus, middle frontal gyrus, and the dorsal ACC) and the posterior left parietal cortex (Figure 3).

### Nucleus Accumbens as a Secondary Seed of Interest

By employing the nucleus accumbens as a secondary seed of interest, we confirmed strong similarities in the changes in functional connectivity observed in the leptin repletion comparison for the hypothalamus and nucleus accumbens. Specifically, like the right hypothalamus, the bilateral nucleus accumbens showed increased functional connectivity with the insula/operculum and the dorsal visual areas (Table S1 and Figure S2) and decreased functional connectivity with the OFC (Table S1 and Figure S3).

## Discussion

We studied neuronal functional connectivity of the hypothalamus before and after weight loss and with and without leptin repletion following weight loss in a group of overweight subjects in an in-patient setting that rigorously controlled variables that have been shown to affect fMRI studies of food intake (e.g. macronutrient content, exercise and the social environment that food is administered) [17]–[19]. We hypothesized that changes in the functional connectivity of the hypothalamus with other brain areas would reflect changes in connectivity that may mediate the observed difficulty with appetite restraint during reduced-weight maintenance and the improved appetite restraint during reduced-weight maintenance with leptin repletion. The major findings of this study are, in response to viewing food cues: 1.) during reduced-weight maintenance, the functional connectivity of the hypothalamus increases with visual and attention areas, 2.) during leptin repletion following weight loss, the functional connectivity of the hypothalamus with the insula and the central and parietal operculae is increased and the functional connectivity of the hypothalamus with the OFC, frontal pole, and dorsal ACC is decreased, which indicate a possible reintegration of hypothalamic functional circuitry with certain frontal reward valuation and emotion processing areas. Taken together, these data are consistent with a mechanism of up-regulation of neural sensitivity to food cues following weight loss that is partially reversed by leptin repletion.

### Reduced-weight Maintenance Comparison

In the reduced-weight maintenance comparison (Wt_−10%placebo_ contrasted with Wt_initial_), while responding to food cues versus non-food cues, the right hypothalamus was found to increase its functional connectivity principally with attention, executive function, visual and memory systems (Figure 2), i.e. in brain areas that have been implicated in the allocation of attentional and decision making resources [20]. More specifically, the ventral visual stream (including the fusiform gyrus, and the inferior temporal gyrus) is critical in object recognition and the dorsal visual stream (including the inferior parietal cortex, superior LOC and cuneus) is critical in object localization and coordination with the motor cortex when in pursuit of an object [21]. The hippocampus and parahippocampal gyrus, which also increased functional connectivity with the hypothalamus in response to food cues following weight loss, are known to be involved in memory function. The areas found to increase their functional connectivity are consistent with those that showed increased functional activation in response to food versus non food cues in a recent meta-analysis [22]. These findings are also consistent with the subjects’ reports that their hunger increased, their satiety was delayed and their perception of food intake decreased [7]. The neural results reported here for the reduced-weight maintenance, suggest a mechanism by which physiological responses to a food-deprived state include strengthening the functional connectivity of the hypothalamus with attention, visual and memory resources when in the presence of external food cues.

### Leptin Repletion Comparison

The leptin injections given after 10% weight loss were calibrated to return their circulating leptin plasma concentrations to the same levels as before weight loss (i.e. leptin repletion). As a result of the leptin repletion, there was an increase in functional connectivity of the right hypothalamus with interoceptive and right dominant visual areas (Figure 2) and decreased functional connectivity with right reward and attention-related areas (Figure 3).

Specifically, we observed increased functional connectivity between the hypothalamus and the right mid- and posterior insula and the associated right central and parietal operculae (Figure 2), which are known to be related to interoception (including hunger) and gustation. [23]–[25]. The mid- and posterior regions of the insula are centrally positioned between the temporal, frontal and parietal cortices and as such well positioned for integrative interoceptive monitoring and processing. One recent study, which used a multi-modal convergent approach to mapping the insula in conjunction with an overlay from a behavior meta-analysis, identified the specificity for interoception with the insula [24]. Another recent functional connectivity study of the insula, identified two distinct right mid-insula areas as subserving interoception, a ventral one centered at z = 0 and a dorsal centered at z = 16 [25]. Our findings did encompass both of these locations but did not detect differences between them, which could be due to the increased size of our smoothing kernel (8 mm) as compared to theirs (6 mm).

The observation that leptin repletion is associated with increases in functional connectivity with these interoceptive areas suggests a recoupling of homeostatic regulating mechanisms including the hypothalamus with the interoceptive monitoring and processing areas that were disassociated following weight loss. This interpretation is further supported by findings both in our single condition results and in other fMRI studies that examined reward activation. First, in our single condition result of Wt_−10% placebo_ no significant functional connectivity is observed between the right hypothalamus and the insula (bilaterally) or the operculae (Figure S4) yet after leptin repletion the functional connectivity is significantly increased (Figure 3).

Second, decreased connectivity of the right hypothalamus, relative to viewing food cues during leptin repletion, is centered on the orbital frontal cortex, the frontal pole, the attentional system and the dorsal visual system (Figure 3). The orbital frontal cortex has been shown to project to the lateral hypothalamus [26] and identified as a primary reward valuation location with various dissociations between lateral and medial areas [27]–[29]. Our findings indicate decreased functional connectivity from the right hypothalamus to the lateral and mid-lateral reaches of the OFC and frontal pole. Two previous fMRI studies have shown that the lateral OFC responds to changes in the relative reward valuation. One showed that the lateral OFC response declines when the subject is sated [28]. The other demonstrated that the lateral OFC responds parametrically to changes in positive reward values [30]. Our observation of a decrease in the functional connectivity of the hypothalamus with the right lateral OFC, during leptin repletion extends these results and suggests that leptin repletion weakens the ability of the lateral OFC to project reward values to the hypothalamus. Thus, the decreased connectivity shown here suggests a mechanism by which leptin repletion decreases coupling between the right hypothalamus and the frontal reward areas resulting in reduced hunger and increased satiety [7]. Similarly, the concomitant weakening of the functional connectivity of the hypothalamus with the attentional (dorsal ACC) and visual systems (superior LOC and inferior parietal lobule) in response to food cues during leptin repletion is consistent with this mechanism (Figure 3).

### Other Functional Connectivity Research in Obese Populations

To our knowledge only two previous studies examined functional neural circuits in obese subjects. Both studies compared obese to normal subjects rather than the within subject comparison of this study. Neither of the previous studies included the hypothalamus as a seed nor included a reduced-weight maintenance comparison or a treatment intervention.

In one study [31] brain responses to viewing high calorie v. low calorie food images were compared between 12 obese subjects and 19 normal-weight subjects. The connectivity test used was not PPI, rather, a form of path analysis was used that tests a specific *a priori* model of the directionality of connection in a pre-specified functional network. Their DCM model focused on differences in functional connections between the OFC, the amygdala and the nucleus accumbens. It was reported that the orbital frontal cortex of the obese subjects compared to lean subjects strongly modulated the nucleus accumbens. Our finding that leptin repletion reduced the functional connectivity between the OFC and both the hypothalamus and nucleus accumbens, reflects and extends their finding for a within-subject study where patients adapt to either weight loss with leptin or weight loss without leptin.

In the other study [32], functional activation was used to locate the caudate nucleus as its seed. The study used PPI and reported a decreased functional connection from the caudate nucleus to the amygdala and insula in the obese subjects compared to the normal-weight subjects, when viewing appetizing v. bland food. As Figure S3 indicates, in the leptin repletion comparison, we observed a decrease of functional connectivity between the nucleus accumbens and both the right anterior insula and the left amygdala during leptin repletion, which is the condition most comparable to the physiological state of homeostasis in obesity.

The findings from our study expand those above in three ways. First, our with-in subject cross-over design highlight brain connectivity changes that can be attributed specifically to the dynamics of weight-loss maintenance within the clinically important obese population. Second, by using PPI without an *a priori* model our technique is well suited to exploratory research in a little studied field. And third, our specific results indicate newly observed changes in functional connectivity between the hypothalamus and reward and attention areas, in response to food cues, that are worthy of further investigation.

### Conclusion

We observed variations in the functional connectivity of the hypothalamus during reduced-weight maintenance and leptin repletion. During reduced weight maintenance, functional connectivity of the hypothalamus increased to attentional, visual, control and reward-sensitive brain areas when viewing food relative to non-food cues suggesting a higher sensitivity to food cues and rewards by the obese during weight loss. During leptin repletion, we observed increased connectivity between the hypothalamus (and the nucleus accumbens) and the insula and operculae, suggesting a possible reintegration of some energy intake regulation mechanisms of the hypothalamus with the interoceptive monitoring of the insula. Finally, during leptin repletion, we also observed a decreased functional connectivity between the hypothalamus (and nucleus accumbens) with the lateral OFC, suggesting a down regulation of reward valuation signals reaching the hypothalamus. Taken as a whole and in conjunction with other recent studies, our findings are consistent with the hypotheses that the functional neural circuits engaged during reduced-weight maintenance biases behavior toward food intake and that during leptin repletion the functional neural circuits bias toward a normalization of appetitive drive and behavior, when exposed to food cues. It is also important to note that leptin repletion impacts the functional connectivity of many areas not directly reflected in our hypotheses.

### Limitations

Despite having the advantage of a carefully controlled weight-loss and leptin repletion study protocol, several limitations to our findings are to be noted. First, during the entire study period, the subjects remained on a synthetic liquid diet and our stimulus set included the presentation of real food items. Thus neural responses of the subjects may also have represented interactions with long-term memory and other functional circuits as they reacted to the presence of real foods during scanning sessions. Second, several studies have shown modest structural differences in the brains of obese individuals. Our registration and identification of the functional network relied on the MNI-152 template, which is an average of 152 young men from the general population. This may limit the accuracy of our identification and localization of some brain structures. Third, two subjects did not have standard structural images of their brain available. This may also have limited the precision of our localization of brain structures. Fourth, the hypothalamus is a relatively small brain structure and is infrequently localized in brain imaging. Spatial smoothing of 8 mm likely limits the localization of each subject’s hypothalamus, though our two-step seed localization process may partially help offset this (see Methods). Fifth, that leptin also acts directly on the ventral tegmental area [33] and can up-regulate its dopaminergic projections may play a role in indirect frontal cortex connectivity changes observed here and it is not possible to dissociate the direct effect of leptin from the indirect effect. Sixth, numerous studies suggest that the effects of leptin on energy homeostasis in humans are dependent upon the nutritional milieu in which leptin is administered [5], [8], [34]–[40]. Thus, the results of this study cannot be assumed to reflect the actions of leptin in any context other than reduced-weight maintenance, solely through caloric restriction. Seventh, our subject sample size is relatively small (n = 10) and no healthy control sample was compared to our weight reduced obese sample. Because our study protocol was a crossover design, each subject served as their own control. Eighth, this study examines the effects of short-term (5 weeks) leptin repletion on PPI in obese weight-reduced subjects following dietary weight loss. These results cannot be generalized to the possible efficacy of longer term leptin repletion. The observations that adaptive thermogenesis persists even many years after weight loss [41] and the long-term efficacy of leptin repletion in leptin deficient subjects on both energy expenditure and intake [42], [43] suggests that exogenous leptin administration following weight loss might be a useful adjunctive therapy, although this hypothesis remains untested. Finally, our choice of the right hypothalamus as our hypothalamic seed rather than the bilateral hypothalamus is based on prior GLM observations from a portion of this data set [8] although we have no *a priori* hypothesis with regard to hypothalamic laterality.

## Methods

### Subjects

Ten obese (BMI >30) subjects (2 male, 8 female) remained as in-patients in the General Clinical Research Center at Columbia University Medical Center throughout the study. All subjects had been stable at their maximal lifetime weights for at least 6 months prior to admission, were in good health, were taking no medications and were right-hand dominant. The study was approved by the Institutional Review Board of The New York Presbyterian Medical Center and are consistent with guiding principles for research involving humans [44]. Written informed consent was obtained from all subjects. Subject characteristics are presented in **Table 2** **.** Six of the 10 subjects included in this fMRI functional connectivity study were also included in our prior functional activation study (8).

**Table 2:** Subject Characteristics (n = 10, 8 females).

	Wt_initial_		Wt_−10%placebo_		Wt_−10%leptin_	
	Mean	SD	Mean	SD	Mean	SD
Age at admission	36.8	6.5				
Weight (Kg)	109.4	26.6	95.4*	23.9	94.1*	24.1
BMI (kg/m^2^)	39.9	8.2	34.8	7	34.3	6.7
Fat Free Mass (Kg)	58.2	14.9	53.0*	12.9	52.2*	15.2
Fat Mass (Kg)	51.2	15.2	42.4*	14.9	41.9*	14.3
Leptin (ng/ml)	50.5	28.5	37.8*	24.7	61.7†	31.3

* p<0.001 vs. Wt_initial_;

† p<0.001 vs. Wt_−10%placebo._

### Study Protocol

See Figure S1 for a diagram of the study design. Prior to the initial scanning session, subjects were fed a liquid formula diet (40% of calories as fat [corn oil], 45% as carbohydrate [glucose polymer], and 15% as protein [casein hydrolysate]), plus vitamin and mineral supplements, in quantities sufficient to maintain a stable weight for six to eight weeks. The initial stabilized weight plateau was designated the Wt_initial_ condition. Following completion of the Wt_initial_ imaging session (described below), subjects were provided 800 kcal/d of the same liquid formula diet until they had lost 10% of their Wt_initial_ weight. The duration required to lose the 10% of initial weight ranged from five to nine weeks. Once 10% weight loss was maintained for six weeks, caloric intake was adjusted upward until subjects were again weight stable at Wt_initial_ less 10% and then remained on an isocaloric diet through the remainder of the study. Subjects then participated in a crossover design of two five-week treatment conditions. During the Wt_−10%placebo_ treatment condition the subjects received s.c. injections of saline. During the Wt_−10%leptin_ condition the subjects received s.c. injections of recombinant methionyl human leptin (provided by Amylin Pharmaceuticals Inc.). The leptin dose was calibrated to re-establish circulating leptin concentrations equal to those measured at Wt_initial_. The order of the treatment conditions was randomly assigned for each subject. Between their two treatment conditions, each subject underwent a 2-week washout period during which they received no injections. During the two treatment conditions and the wash-out phase, subjects were unaware of the order of treatments and remained on the isocaloric diets that previously maintained their weight at the Wt_initial_ less 10%–12%.

### Image Acquisition

Images were acquired on a General Electric 1.5T scanner. Functional images were acquired with a T2-weighted echo planar imaging (EPI) sequence, using a TR (time to repeat) of 4,000 ms, an echo time of 60 ms, a flip angle of 60°, a field of view of 190 mm×190 mm with an array size of 128×128). Twenty-five contiguous 4.5-mm-thick axial slices were acquired parallel to the anterior-posterior commissure. The resulting functional voxel size was 1.5 mm×1.5 mm×4.5 mm. Structural images were acquired with a T1-weighted spoiled gradient–recalled (SPGR) sequence using a TR of 19 ms, an echo time of 5 ms, a flip angle of 20° a field of view of 220 mm×220 m, recording 124 slices at a thickness of 1.5 mm. The resulting structural voxel size was.86 mm×0.86 mm×1.5 mm. Structural T1 images were not available for two subjects. The mean high-resolution functional image for these two subjects was used in lieu of the structural T1 for registration purposes (see below).

### Functional Scanning Sessions and Stimulus Presentation

Scanning sessions for each of the three conditions were identical and occurred in a post-absorptive state beginning at approximately 9 am. Four functional scans of 36 acquisitions were collected, each lasting 2 minutes 24 seconds. Visual stimuli were the same as used in our prior functional activation study (8). In two functional scans 12 food items (e.g. fruit, grains, vegetables, sweets) were visually presented for four seconds each in a single block that was preceded and followed by a baseline. In the other two scans 12 non-food items (e.g. cell phone, jump rope, yo-yo) were visually presented in a similar manner. See Table S2 for a list of the stimuli. The order of stimuli presentation was randomly assigned *a priori* but was retained for each subsequent scanning session for each subject.

### Image Preprocessing

All preprocessing and statistical analyses were completed using the FMRIB Centre’s FSL FEAT version 5.98 [45]. Following image reconstruction and the deletion of the first three acquisitions, the data for each functional scan was slice time corrected, spatially smoothed at 8 mm FWHM, high-pass filtered at 100 seconds and motion corrected using McFLIRT. The functional and structural scans for eight subjects were co-registered using six degrees of freedom and the result was co-registered to the Montreal Neurological Institute-152 brain template (voxel size, 2 mm^3^) using 12 degrees of freedom.

### PPI Analysis

Psychophysiological interaction (PPI) analysis [12] was completed to examine changes in functional connectivity of neural circuits when responding to food stimuli compared to non-food stimuli. The PPI analysis was completed as a linear univariate regression within the Generalized Linear Model framework of FEAT [46]. Given a region of interest seed in the brain, PPI analysis identified brain areas with activation more highly correlated during one condition than another. For each functional scan the regressor of interest (PPI) was constructed as the scalar product of the psychological regressor (the time course of the stimuli presentations convolved with a double gamma hemodynamic response function) and the physiological regressor (the activation time course of the seed). Nuisance regressors included the psychological regressor, the physiological regressor, a global mean regressor, a white matter regressor, and for further motion correction three translation and three rotation regressors. No CSF regressor was used because of the hypothalamus’ proximity to the fossa. Voxel level results were thresholded at a probability 0.05 and cluster-level corrections were made for multiple comparisons in the regression using Gaussian Random Field Theory with a probability for each cluster of 0.01.

Prior to testing the specific hypothesis, we first tested for a presence of functional networks within each of the three individual treatment conditions: Wt_initial_, Wt_−10%placebo_ and Wt_−10%leptin_. Then, to test the reduced-weight maintenance hypothesis, we tested for significant changes by contrasting the Wt_−10%placebo_ condition with the Wt_initial_ condition. To test the leptin repletion hypothesis, we tested for significant changes by contrasting the Wt_−10%leptin_ condition with the Wt_−10%placebo_ condition. The above sequence of tests was repeated for both seeds.

### PPI Seeds of Interest

Since the hypothalamus is a primary mediator of hormonal signaling regarding hunger and satiety, it was the primary seed of interest. The hypothalamus is thought to be the central locus for homeostatic feeding control [47] and is the primary brain site for direct leptin action [48]. Further, the hypothalamus was recently shown to modulate non-feeding behaviors by up-regulating dopamine (DA) projecting neurons in the ventral tegmental area (VTA) when hypothalamic agouti-related protein (AgRP) cells are impaired [49]. Moreover, in our prior study, the right hypothalamus presented a robust response to the leptin treatment.

In addition, the nucleus accumbens was chosen as a secondary seed because it has a central role in hedonic motivation (including relative to food), is an indirect recipient of leptin action from leptin sensitive neurons in the ventral tegmental area via dopaminergic projections [33], [50]–[52], is directly connected multi-synaptically to the both the arcuate nucleus of the hypothalamus and the lateral hypothalamus [53], and is reciprocally connected by hypothalamic projections into the shell of the nucleus accumbens/ventral striatum [54], which in turn projects to the dorsal striatum (putamen), where direct initiation and termination of behavior is observed. The results from the nucleus accumbens seed were used to provide additional support for our leptin repletion findings relative to the hypothalamic seed.

The seeds for both the hypothalamus and the nucleus accumbens were constructed in a two-step process. We started with an externally determined group seed mask and then re-centered within that location based on the maximum activation of the individual subject within that group mask. Specifically, the location for the initial hypothalamic group mask was taken from the activation location reported for the right hypothalamus in our previous study [8]. Around this location a spheroid was constructed using a 4 mm kernel. The resulting spheroid was then tested for the voxel of maximal activation for each subject. Around this re-centered voxel, the final seed for the hypothalamus was constructed as a new spheroid for each subject using a 6 mm kernel. For the nucleus accumbens, we started with a group mask derived from all voxels having a 50% or greater probability of being identified as nucleus accumbens by the probabilistic Oxford-Harvard Subcortical Structural Atlas. Within that mask, we then tested for the voxel of maximum activation for each subject. Around this re-centered voxel, the final seed for the nucleus accumbens was constructed as a new spheroid for each subject using a kernel of 6 mm.

## Supporting Information

Figure S1 **Study Design.** Horizontal Axis indicates approximate duration of time in the study and comparisons between conditions. Vertical axis indicates approximate weight. The three conditions of the study Wt_initial_, Wt_−10%placebo_, and Wt_−10%leptin_ are shown in the burnt orange, yellow and light orange rectangles. The comparisons between the three conditions are illustrated in the two double-arrow blue figures. Scanning occurred at the end of each condition and the order of the two reduced-weight conditions was counter balanced [8].
(TIF)

Figure S2 **Increased functional connectivity of the nucleus accumbens in the reduced-weight maintenance with leptin repletion comparison.** Brain areas showing significant increases in functional connectivity with the nucleus accumbens seed (indicated in copper) are shown on standard space axial brain slices with the color indicating the Z score per the color gradient on the bottom. STG (Superior Temporal Gyrus), PCC (Posterior Cingulate Cortex), sup LOC (superior division of the Lateral Occipital Cortex).
(TIF)

Figure S3 **Decreased functional connectivity of the nucleus accumbens in the reduced-weight maintenance with leptin repletion comparison.** Brain areas showing significant decreases in functional connectivity with the nucleus accumbens seed (indicated in copper) are shown on standard space axial brain slices with the color indicating the Z score per the color gradient on the bottom. Med & lat OFG (medial and lateral Orbital Frontal Cortex), ventral ACC (ventral Anterior Cingulate Cortex), Amyg (Amygdala), FP (Frontal Pole).
(TIF)

Figure S4 **Increased functional connectivity in the reduced-weight maintenance with placebo injections condition.** Brain areas showing significantly correlated functional connectivity with the hypothalamic seed (indicated in green) are shown on standard space axial brain slices with the color indicating the Z score per the color gradient on the bottom. Sup LOC (superior division of Lateral Occipital Cortex), dorsal ACC (Anterior Cingulate Cortex), MFG (Middle Frontal Gyrus), SFG (Superior Frontal Gyrus).
(TIF)

Table S1 **Changes in Functional Connectivity.** Seed: Bilateral Nucleus Accumbens.
(DOCX)

Table S2 **Food and Non-Food Stimuli.**
(DOCX)
