# Supplementary figures and images for: Effects of Reduced Weight Maintenance and Leptin Repletion on Functional Connectivity of the Hypothalamus in Obese Humans
Source: PLoS One. 2013 Mar 21;8(3):e59114. doi: 10.1371/journal.pone.0059114 (PMC3605420; doi:10.1371/journal.pone.0059114)

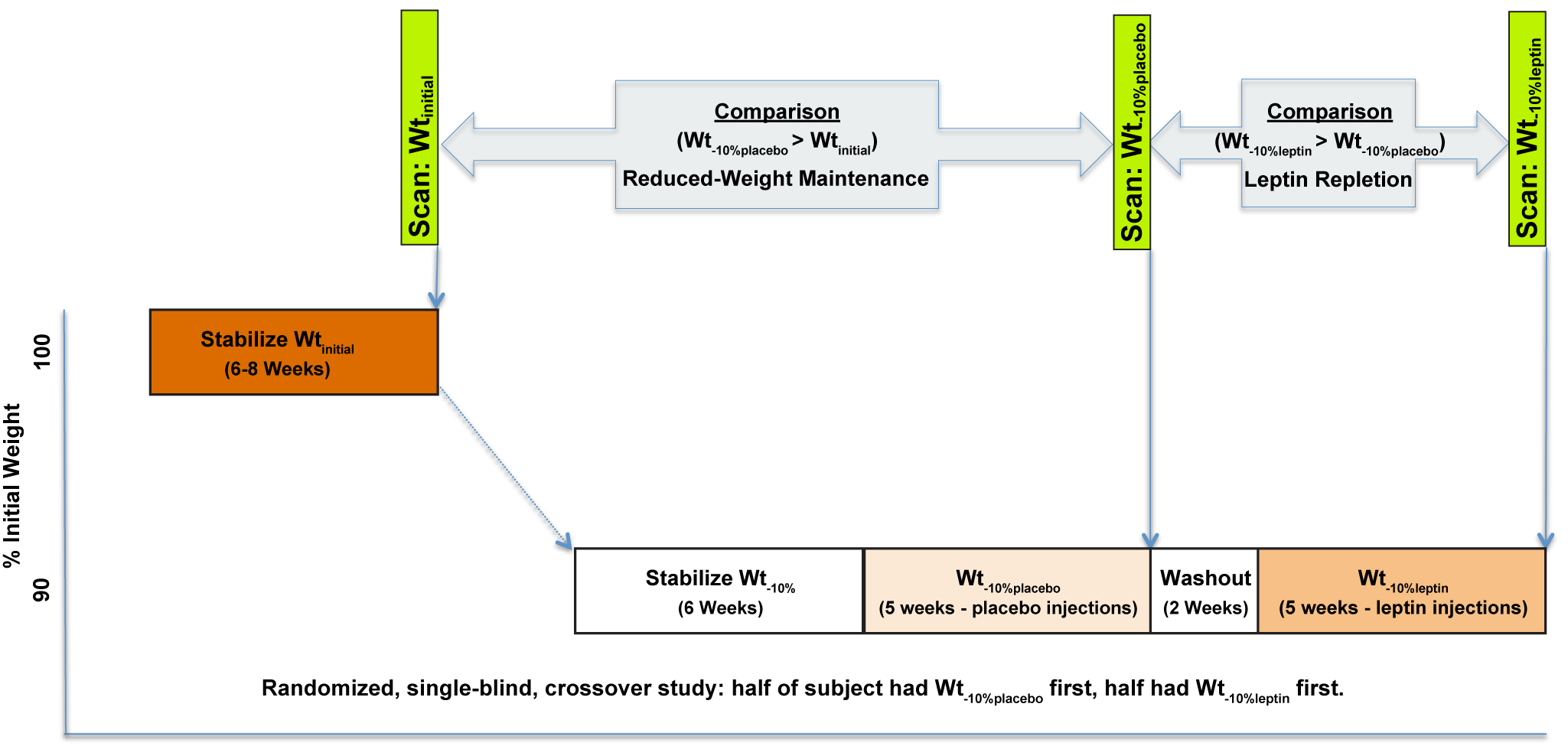

Supplement: Figure S1 — Study Design. Horizontal Axis indicates approximate duration of time in the study and comparisons between conditions. Vertical axis indicates approximate weight. The three conditions of the study Wtinitial, Wt−10%placebo, and Wt−10%leptin are shown in the burnt orange, yellow and light orange rectangles. The comparisons between the three conditions are illustrated in the two double-arrow blue figures. Scanning occurred at the end of each condition and the order of the two reduced-weight conditions was counter balanced [8]. (TIF) [file pone.0059114.s001.tif]

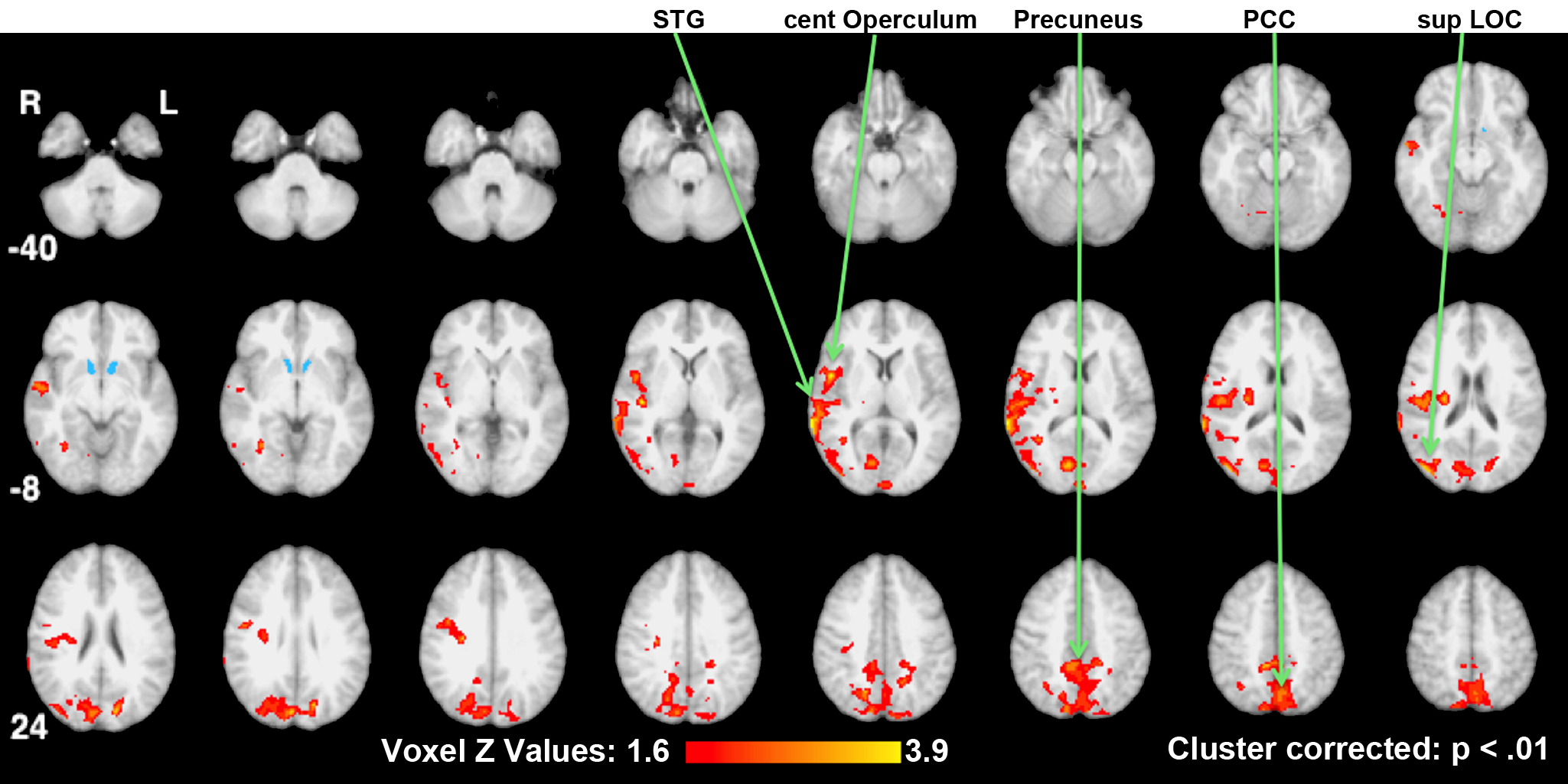

Supplement: Figure S2 — Increased functional connectivity of the nucleus accumbens in the reduced-weight maintenance with leptin repletion comparison. Brain areas showing significant increases in functional connectivity with the nucleus accumbens seed (indicated in copper) are shown on standard space axial brain slices with the color indicating the Z score per the color gradient on the bottom. STG (Superior Temporal Gyrus), PCC (Posterior Cingulate Cortex), sup LOC (superior division of the Lateral Occipital Cortex). (TIF) [file pone.0059114.s002.tif]

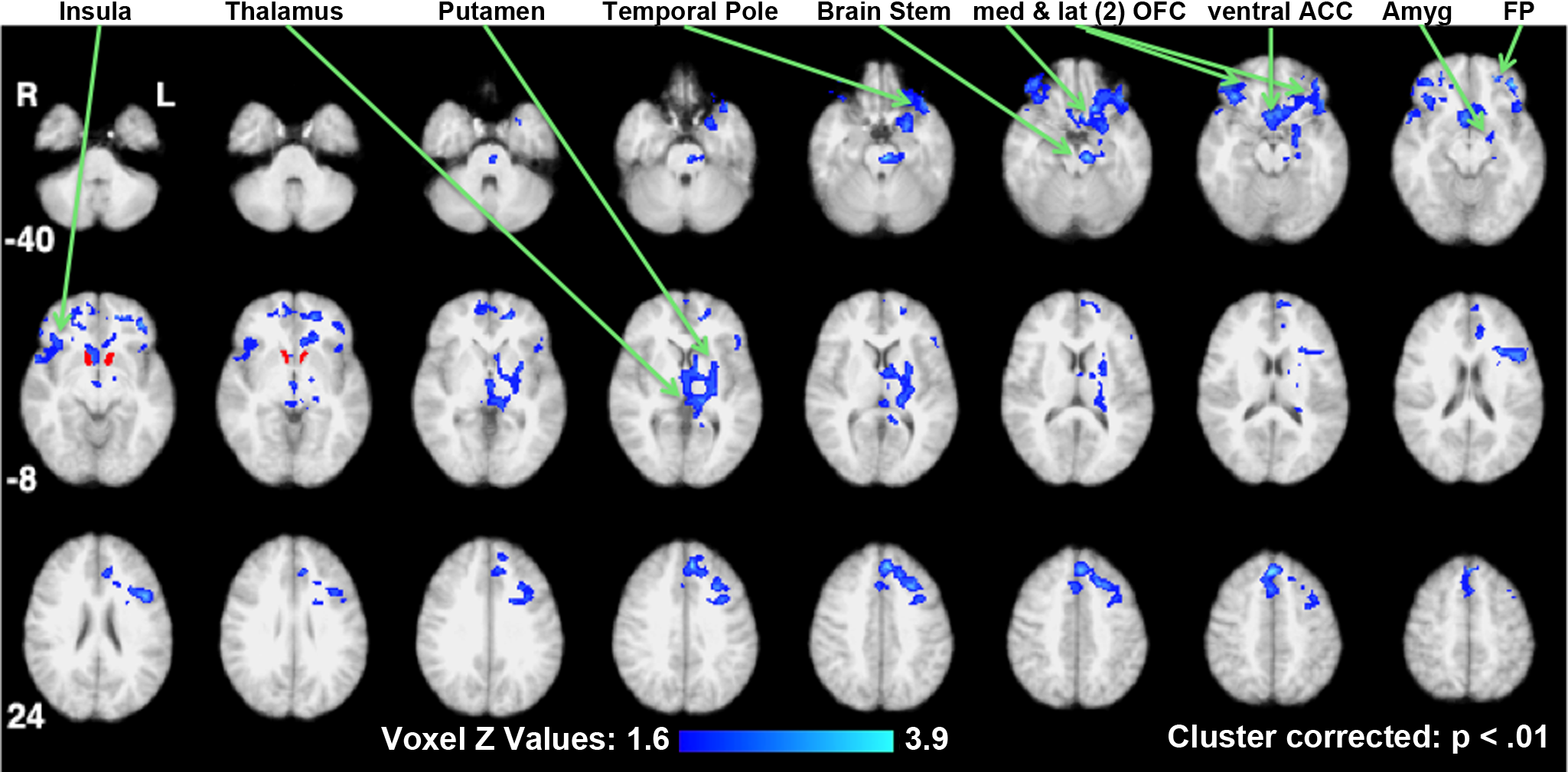

Supplement: Figure S3 — Decreased functional connectivity of the nucleus accumbens in the reduced-weight maintenance with leptin repletion comparison. Brain areas showing significant decreases in functional connectivity with the nucleus accumbens seed (indicated in copper) are shown on standard space axial brain slices with the color indicating the Z score per the color gradient on the bottom. Med & lat OFG (medial and lateral Orbital Frontal Cortex), ventral ACC (ventral Anterior Cingulate Cortex), Amyg (Amygdala), FP (Frontal Pole). (TIF) [file pone.0059114.s003.tif]

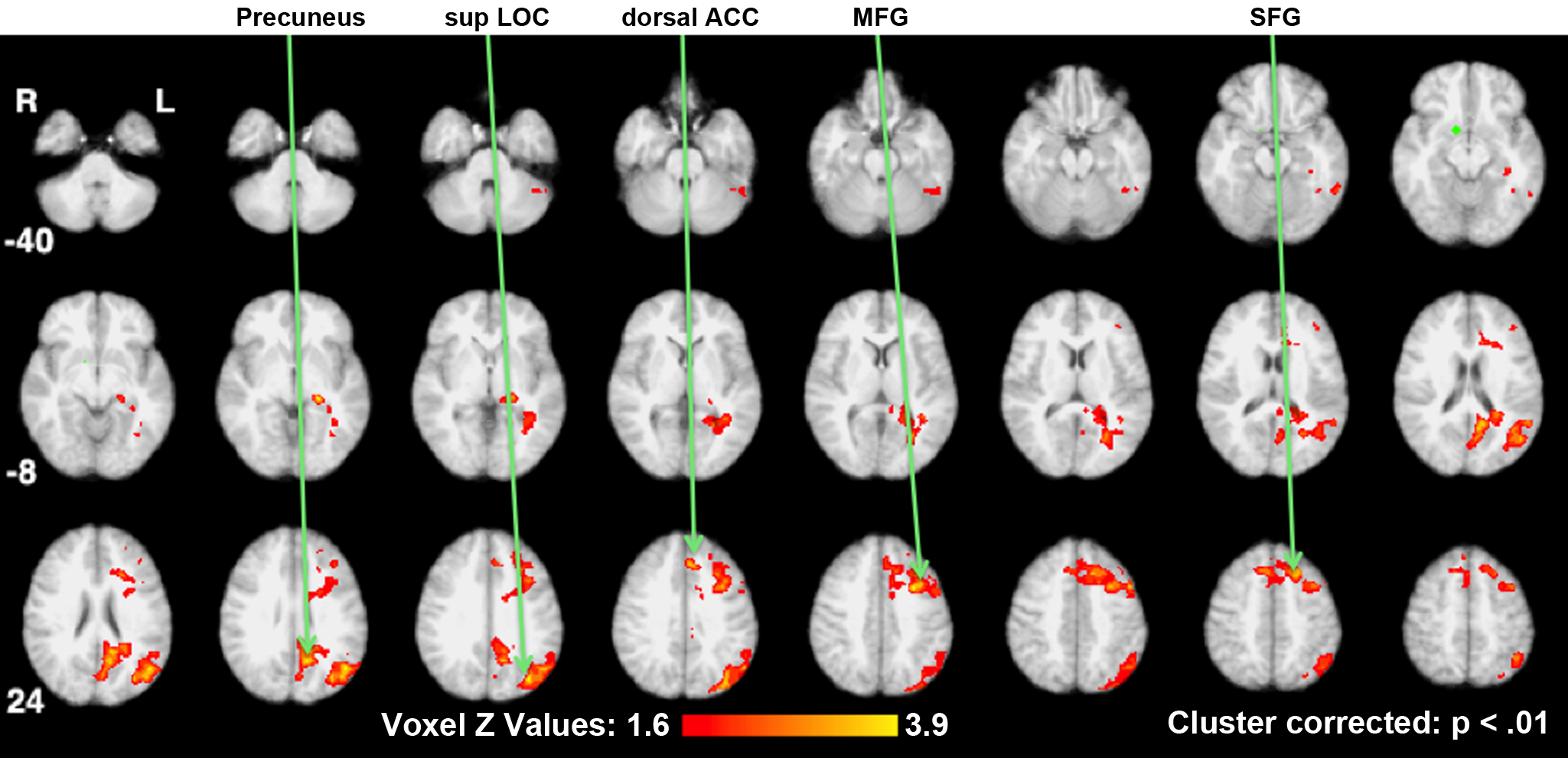

Supplement: Figure S4 — Increased functional connectivity in the reduced-weight maintenance with placebo injections condition. Brain areas showing significantly correlated functional connectivity with the hypothalamic seed (indicated in green) are shown on standard space axial brain slices with the color indicating the Z score per the color gradient on the bottom. Sup LOC (superior division of Lateral Occipital Cortex), dorsal ACC (Anterior Cingulate Cortex), MFG (Middle Frontal Gyrus), SFG (Superior Frontal Gyrus). (TIF) [file pone.0059114.s004.tif]
