# Supplementary material for: Effects of Reduced Weight Maintenance and Leptin Repletion on Functional Connectivity of the Hypothalamus in Obese Humans
Source: PLoS One. 2013 Mar 21;8(3):e59114. doi: 10.1371/journal.pone.0059114 (PMC3605420; doi:10.1371/journal.pone.0059114)
Supplement: Table S1 — Changes in Functional Connectivity. Seed: Bilateral Nucleus Accumbens. (DOCX) [file pone.0059114.s005.docx]

| **Table S1: Changes in Functional Connectivity^1^** | | | | | | |
| --- | --- | --- | --- | --- | --- | --- |
| **Seed: Bilateral Nucleus Accumbens** | | | | | | |
| **Comparison** | | **Structure** | **Z score** | **x** | **y** | **z** |
| **Leptin Repletion (Wt_-10%leptin_ > Wt_-10%placebo_)** | | |  |  |  |  |
|  | **(See Fig. S1)** | Significant **increased** functional connectivity relative to food cues: | | | |  |
|  |  | Superior Temporal Gyrus (STG) | 3.95 | 68 | -38 | 8 |
|  |  | superior Lateral Occipital Cortex | 3.88 | 44 | -80 | 18 |
|  |  | central Operculum | 3.65 | 50 | 4 | 8 |
|  |  | Superior Temporal Gyrus (STG) | 3.56 | 60 | -32 | 10 |
|  |  | Precuneus | 3.40 | 18 | -64 | 40 |
|  |  | PCC | 2.33 | 10 | -38 | 42 |
|  | **(See Fig. S2)** | Significant **decreased** functional connectivity relative to food cues: | | | |  |
|  |  | Brain Stem | 3.92 | -8 | -22 | -22 |
|  |  | Frontal Pole | 3.56 | -40 | 42 | -12 |
|  |  | med OFC | 3.41 | -16 | 20 | -20 |
|  |  | lat OFC | 3.31 | -40 | 42 | -8 |
|  |  | Temporal Pole | 3.28 | -40 | 26 | -26 |
|  |  | lat OFC | 3.18 | 36 | 32 | -16 |
|  |  | ventral ACC | 2.88 | 2 | 14 | -12 |
|  |  | Putamen | 2.39 | -26 | -6 | 4 |
|  |  | Thalamus | 2.36 | -10 | -32 | 4 |
|  |  | Amygdala | 2.36 | -22 | -8 | -16 |
|  |  | Insula | 1.79 | 42 | 16 | -8 |
| ^1^ Located as local maxima in clusters expressed in the Montreal Neurological Institute 152 brain template. | | | | | | |
|  | | | | | | |
