# Supplementary material for: Effects of Reduced Weight Maintenance and Leptin Repletion on Functional Connectivity of the Hypothalamus in Obese Humans
Source: PLoS One. 2013 Mar 21;8(3):e59114. doi: 10.1371/journal.pone.0059114 (PMC3605420; doi:10.1371/journal.pone.0059114)
Supplement: Table S2 — Food and Non-Food Stimuli. (DOCX) [file pone.0059114.s006.docx]

| \| **Table S2: Food and Non-Food Stimuli** \| \| \| \| \| --- \| --- \| --- \| --- \| \| **Foods** \| \| **Non-Foods** \| \| \| apple \| hershey kisses \| bags \| pens \| \| bagel \| Lemons \| bracelets \| q-tips \| \| bananas \| Loaf \| candles \| rubber balls \| \| broccoli \| Lollipops \| cds \| rubber bands \| \| carrots \| Muffin \| cellphone \| scarves \| \| celery \| Nuts \| comb \| scotch tape \| \| cheese \| Oreos \| dolls \| soap \| \| corn \| potato chips \| jump rope \| sponge \| \| crackers \| Pretzels \| kleenex \| stuffed animal \| \| granola bars \| sliced bread \| marbles \| sunglasses \| \| grapes \| Twizzlers \| matches \| tennis balls \| \| gummi worms \| wrapped candies \| notepad \| toothbrush \| |
| --- | --- | --- | --- | --- | --- | --- | --- | --- | --- | --- | --- | --- | --- | --- | --- | --- | --- | --- | --- | --- | --- | --- | --- | --- | --- | --- | --- | --- | --- | --- | --- | --- | --- | --- | --- | --- | --- | --- | --- | --- | --- | --- | --- | --- | --- | --- | --- | --- | --- | --- | --- | --- | --- | --- | --- | --- |
|  |
